# A new partnership with *Journal of Experimental Botany*

**DOI:** 10.1002/pld3.353

**Published:** 2021-09-21

**Authors:** Ivan R. Baxter

**Affiliations:** ^1^ Donald Danforth Plant Science Center St. Louis Missouri USA

Two of our goals when we started *Plant Direct* were to make publishing plant research easier and to become the sound science society journal of the plant sciences. One of the ways we have tried to accomplish these goals is to take papers transferred from our partner journals: *Plant Physiology*, *The Plant Journal*, and *The Plant Cell*. Transferring papers saves the authors' time and effort in filling out the meta data associated with each submission—but the real benefits come when the transfers include reviews from the partner journal. With transferred reviews, we can save the authors' and reviewers' time and prevent future rounds of reviews. From *Plant Direct*'s perspective, the more transfers we can get, the better we are able to fulfill our sound science mission and ensure that all good work can find a home in a society journal.

So I am very excited to announce that we have added a new partner journal: *The Journal of Experimental Botany* (*JExpBot*) from one of our Societies—the Society of Experimental Biology. The goal of *JExpBot* is to publish papers “that advance our understanding of plant biology”—a perfect fit for *Plant Direct*. Authors are now able to port manuscripts declined at *JExpBot* to *Plant Direct* with the click of a button. We are looking forward to our partnership!

